# Investigation of prospective effects of emotion-regulation difficulties and empathic dimensions on depressive symptoms during the COVID-19 outbreak in poland

**DOI:** 10.1192/j.eurpsy.2021.683

**Published:** 2021-08-13

**Authors:** M. Gambin, M. Woźniak-Prus, M. Sękowski, P. Holas, A. Wnuk, T. Oleksy, A. Cudo, K. Hansen, M. Huflejt-Łukasik, A. Łyś, J. Gorgol, K. Kubicka, G. Kmita, E. Łojek

**Affiliations:** 1 Department Of Psychology, University of Warsaw, Warsaw, Poland; 2 Institute Of Psychology, The Maria Grzegorzewska University, Warsaw, Poland; 3 The Robert Zajonc Institute For Social Studies, University of Warsaw, Warsaw, Poland; 4 Faculty Of Social Sciences, The John Paul II Catholic University of Lublin, Lublin, Poland

**Keywords:** emotion regulation, empathy, depressive symptoms, COVID-19 pandemic

## Abstract

**Introduction:**

During the COVID-19 pandemic people experience higher levels of negative emotions, as well as face many negative and intense emotions felt by others. Thus, it is important to look for risk and protective factors that allow and help individuals to regulate these negative emotions and adapt to the hardships of the COVID-19 pandemic.

**Objectives:**

The main aims of the study were to (i) test how empathic dimensions (perspective taking, empathic concern and personal distress) and emotion regulation abilities were related to intensity of depressive symptoms during the COVID-19 lockdown in Poland, as well as to (ii) check if emotion regulation difficulties and personal distress predicted slower decrease in depressive symptoms over the two months in which the number of COVID-19 cases declined in Poland.

**Methods:**

A total of 792 participants took part in the three-wave panel study. The sample was representative of the Polish population in terms of gender, age, and place of residence. Participants completed the following online questionnaires: The Patient Health Questionnaire-9, The Difficulties in Emotion Regulation Scale Short Form, and Brief version of the Empathic Sensitivity.

**Results:**

Significant positive correlations were found between depressive symptoms and both personal distress and emotion regulation difficulties during the lockdown. Moreover, emotion regulation difficulties were the only significant predictor of slower decrease in depressive symptoms over time during the COVID-19 pandemic.

**Conclusions:**

It seems that interventions focused on improvement of emotion regulation abilities could be particularly beneficial in reducing depressive symptoms during the pandemic and preventing potential negative long-term outcomes.

